# Significant Response to Camrelizumab Plus Targeted Drugs in Recurrent Intrahepatic Cholangiocarcinoma: a Case Report and Literature Review

**DOI:** 10.1007/s12029-021-00637-7

**Published:** 2021-07-26

**Authors:** Peixin Huang, Yingting Zhou, Yi Chen

**Affiliations:** 1 Department of Hepatic Oncology, Zhongshan Hospital, Shanghai, China; 2 Liver Institute, Zhongshan Hospital, Shanghai, China

**Keywords:** Immune checkpoint inhibitors, Camrelizumab, Targeted therapy, Recurrent intrahepatic cholangiocarcinoma

## Abstract

**Purpose:**

Intrahepatic cholangiocarcinoma is the second most common primary liver cancer, and is associated with a poor prognosis and rising incidence rate.

**Methods:**

Here, we reported the case of a middle-aged Asian male who presented with a 9.5-cm liver lesion and was diagnosed with intrahepatic cholangiocarcinoma.

**Results:**

The patient experienced recurrence three times, twice following radical resection and standard adjuvant chemotherapy and once following camrelizumab plus apatinib, after which the tumor progressed with elevated CA 19.9 level. After tissue biopsy for next-generation sequencing, apatinib was replaced by lenvatinib, and the patient achieved disease control again, with a progression-free survival of 10 months.

**Conclusion:**

Combined immunotherapy and targeted therapy regimens are a promising approach for refractory intrahepatic cholangiocarcinoma. Further well-designed prospective clinical trials are needed to confirm the efficacy and safety. Since intrahepatic cholangiocarcinoma is characterized by high heterogeneity and with complex crosstalk among oncogenic pathways, further exploration is required to more deeply understand the mechanism of action of this treatment approach and guide individualized treatment selection.

## Introduction

Cholangiocarcinoma (CCA) is an aggressive liver malignancy with a poor prognosis due to advanced stage at first presentation, drug resistance, and a lack of effective treatment protocols [1]. The incidence of CCA and associated mortality has increased in recent decades [2]. The average 5-year overall survival (OS) for CCA is low, reported as 13–21% for patients with early-stage disease who undergo successful resection [3–6]. According to anatomical location, CCA is subclassified as intrahepatic, perihilar, and distal. Intrahepatic cholangiocarcinoma (iCCA) represents < 10% of all CCA cases [7]. Cirrhosis and viral hepatitis (B and C) have been identified as potential risk factors for CCA, especially iCCA [8, 9].

Surgery is the preferred option for all subtypes of early-stage CCA. Staging laparoscopy in conjunction with surgery is recommended and can help to identify around 25–36% patients unsuitable for open surgery [10]. For patients with unresectable disease, chemotherapy with gemcitabine and cisplatin is considered the standard first-line treatment and is associated with a median OS of < 1 year [11]. However, in second line, there is currently no standard systemic treatment. In clinical practice, locoregional therapies are often used for iCCA, although there is no conclusive evidence supporting their use. Therefore, new systemic treatment options for patients with CCA are an urgent unmet need.

Recently, gene profiling and sequencing techniques have been used to identify patients with CCA harboring genetic aberrations that may predict a good response to targeted therapy and immunotherapy. Combinations of immunotherapy and targeted drugs for the treatment of CCA are being investigated in several ongoing clinical trials (NCT03895970, NCT04361331, NCT04454905, and NCT03779100). Here, we report the case of a 50-year-old Asian male with iCCA who experienced recurrent disease following surgical resection before receiving combined immune checkpoint inhibitors and targeted therapy. The patient achieved a significant response to the combined treatment regimen, with disease control for 20 months at the time of last follow-up. In addition, we conducted a literature review of systemic therapy and new advances in the treatment of CCA.

## Case Presentation

**Fig. 1:**
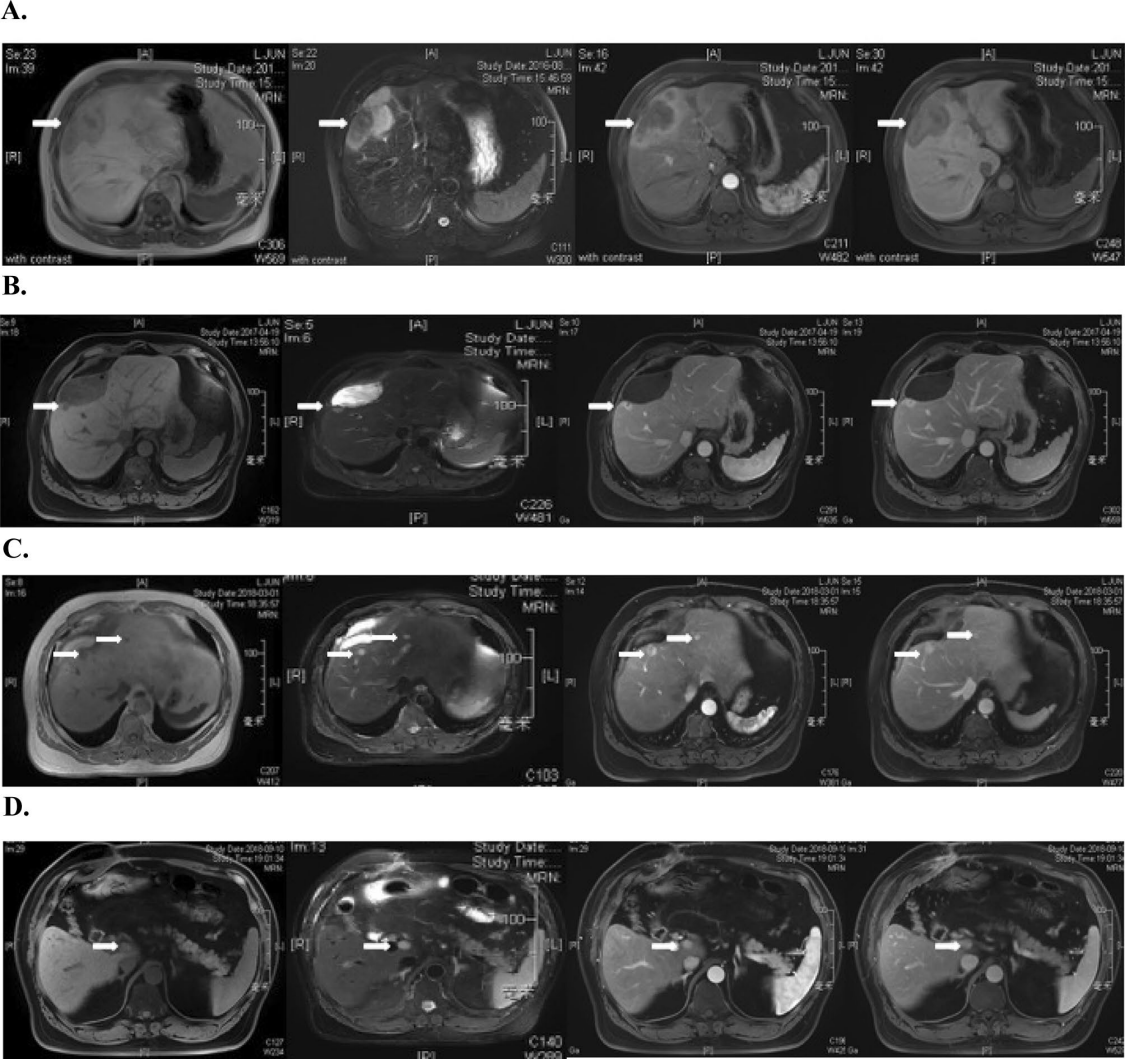
Enhanced abdominal magnetic renounce imaging (MRI) of the reported case. **a** The white arrow heads a hypovascular liver lesion (9.5 cm). **b** The white arrow directs to the recurrent lesion (about 1 cm) adjacent to the first operation zone (1st recurrence). **c** The white arrow shows the recurrent tumors in liver after re-resection (2nd recurrence). **d** The white arrow pointed to the liver lesion after open-surgical microwave ablation (3rd recurrence)

A 50-year-old male was admitted due to a liver lesion and had no history of hepatitis B or C infection and no other medical history. The tumor was detected during an annual health examination. Physical examination and laboratory tests were normal; the patient had Child–Pugh Grade A (Score 5) and was negative for expression of CA 19.9, carcinoembryonic antigen (CEA), and alpha-fetoprotein (AFP). Enhanced abdominal magnetic resonance imaging (MRI) identified a 95 × 52-mm lesion in the liver with enlarged hilar lymph nodes (Fig. 1A).

After discussion with the medical team, the patient accepted meso-hepatectomy, cholecystectomy, and regional lymphadenectomy and underwent the procedures in August 2016. Post-operative histopathological examination showed iCCA with lymph nodes metastasis (3/7), with a negative surgical margin. After surgery, the patient was given six 3-weekly cycles of adjuvant GEMOX chemotherapy (gemcitabine 1000 mg/m^2^ on day 1 and day 8 and oxaliplatin 130 mg/m^2^ on day 1). In April 2017, 8 months after the initial surgery, the patient experienced recurrence after detection of a liver tumor of diameter 1 cm in the right lobe (Fig. 1B). The patient underwent a partial right hepatectomy, and post-surgical pathological evaluation confirmed a diagnosis of iCCA. Immunohistochemistry showed the surgical samples were positive for AFP, CK19, and CD34 and had Ki-67 positivity of 45% but were negative for hepatocytes, glypican-3, and arginase-l. In addition, the tumors were negative for PD-1 and PD-L1, but the tumor stroma showed positivity: PD-1 (tumor, stroma 5%+), PD-L1 [E1L3W] (tumor, stroma 15%+), PD-L1 [SP142] (tumor, stroma 20%+). After recovery from surgery, oral capecitabine (1000 mg/m^2^ per day from day 1 to day 14, every 3 weeks) was initiated and maintained until disease progression.

**Fig. 2:**
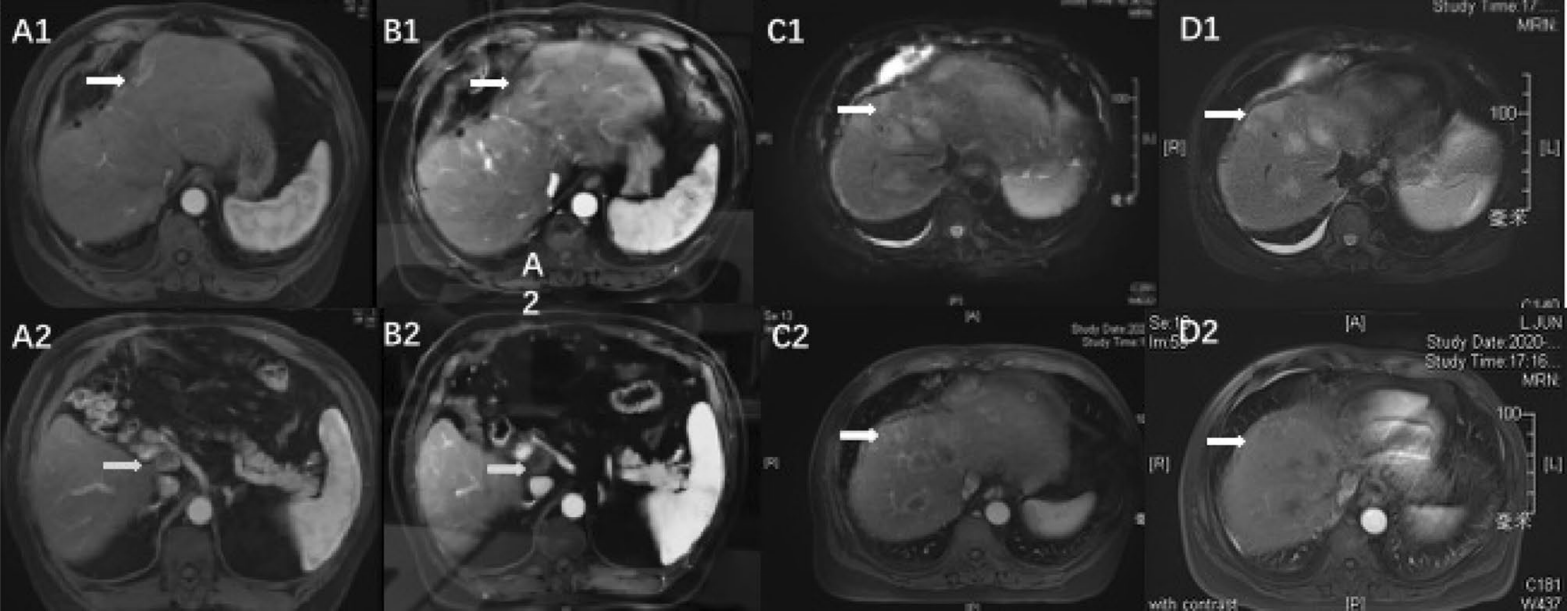
Enhanced abdominal magnetic renounce imaging (MRI) of the reported case. A1, B1 White arrows showed new liver lesion. A2, B2 Cyan arrows direct an enlarged hilar lymph node. C1, C2 The white arrows showed progressed multiple liver lesions. D1, D2 The lesions directed by arrows were evaluated stable by imaging examination after combination therapy

The patient experienced a second recurrence in September 2017, 5 months after the second surgery. Chemotherapy was adjusted to nab-paclitaxel (125 mg/m^2^, on day 1 and day 8) plus gemcitabine (1000 mg/m^2^, on day 1) in 3-weekly cycles for six cycles, and imaging evaluation after chemotherapy revealed stable disease (assessed by RECIST 1.1) in December 2017. However, on March 2, 2018 (3 months later), the patient experienced disease progression, with enlargement of liver lesions (Fig. 1C). Two courses of transarterial chemoembolization (TACE) were performed in March and May 2018. However, new lesions were detected on July 23, 2018. Open-surgical microwave ablation was performed on August 10, 2018. Unfortunately, a new lesion was found near the inferior vena cava on September 14, 2018, 1 month after the third operation (Fig. 1D).

On September 28, 2018, the patient initiated systemic therapy with camrelizumab (200 mg every 2 weeks) combined with apatinib (250 mg orally twice daily). Two weeks later, the dose of apatinib was reduced to 250 mg per day due to fatigue and Grade 3 hypertension. At an imaging evaluation on December 4, 2018, the patient had achieved a complete response (CR) with disappearance of all lesions, which lasted for 8 months.

In June 2019, the camrelizumab dosing interval was adjusted to once every 3 weeks. One month (July 25, 2019) after this dosage adjustment, the patient experienced disease progression; enhanced abdominal MRI showed liver lesions with abdominal lymph nodes metastasis (Fig. 2A). Following disease progression, the dose of camrelizumab was increased to 200 mg every 2 weeks. The subsequent imaging evaluation on November 5, 2019 revealed a reduction in tumor size and necrosis of the lymph node; this response lasted until February 2020 (Fig. 2B). Following the treatment response, a gradual increase in the level of biomarkers CA 19.9 and CEA was observed, and by May 27, 2020, the levels had risen from normal to 89.4 U/ml and 67.5 mg/ml, respectively. Enhanced MRI confirmed increased and enlarged nodules in the liver (Fig. 2C).

An ultrasound-guided biopsy was performed to enable next-generation sequencing (NGS). An isocitrate dehydrogenase 1 (IDH1) mutation was detected, while mismatch repair deficiency (dMMR)/high levels of microsatellite instability (MSI-H) were not detected. The tumor mutation burden (TMB) and PD-L1 expression in the tissue samples were both low. Apatinib was replaced by lenvatinib (8 mg orally once daily) beginning on May 29, 2020 and camrelizumab was continued at 200 mg every 2 weeks. Two months later, imaging evaluation showed reduced tumor enhancement and stable disease, and levels of CA 19.9 and CEA returned to normal (Fig. 2D). At the last follow-up (April 9, 2021), the patient had not experienced disease progression. With immune and targeted therapy, the patient had achieved a progression-free survival of 10 months (315 days) since initiating lenvatinib.

## Discussion

iCCA arises above the second-degree bile ducts within the liver and is divided into five subtypes according to growth patterns: mass-forming, periductal-infiltrating, intraductal, superficial spreading, and undefined. The superficial spreading and intraductal subtypes are associated with the best prognosis and periductal and mass-forming subtypes with the worst [12]. However, overall, the prognosis for patients with iCCA is usually very poor, with average 5-year OS rates between 5 and 10% for patients with unresectable iCCA [13]. The main contributing factors to the poor prognosis include a high recurrence rate and limited efficacy of traditional systemic and locoregional treatments such as chemotherapy and radiotherapy [14].

For patients with unresectable iCCA, clinical trials, systemic therapy, and best supportive care are the primary treatment options. Gemcitabine plus cisplatin is the preferred first-line systemic therapy regimen recommended in most treatment guidelines (NCCN, CSCO, and ESMO). Subsequent-line therapy is usually based around the FOLFOX regimen, supported by the recent ABC-06 study [15]. In ABC-06, nab-paclitaxel combined with cisplatin and gemcitabine showed promising early results in patients with advanced biliary tract cancer (BTC), with a disease control rate (DCR) of 84%, median progression free survival (mPFS) of 11.8 months, and median OS (mOS) of 19.2 months, but these patients experienced significant toxicity [15]. Despite the results from ABC-06, a review of 23 studies (14 phase II clinical trials and 9 retrospective studies) including a total of 761 patients with advanced BTC found insufficient evidence to recommend specific regimens for second-line treatment of CCA [16]. Therefore, more second-line and subsequent treatment strategies are needed. In the present case, the patient received standard adjuvant chemotherapy after the first and second surgical resection. Locoregional therapy (TACE) provides very little benefit.

In the past few years, several advances have been made in the therapeutic approaches for patients with hepatobiliary cancers. Precision medicine requires gene testing before treatment, including MSI/MMR, NTRK (neurotropic tyrosine kinase receptor) gene fusion testing, and/or other molecular testing. According to the results of the MOSCATO-01 trial, actionable molecular targets are detected in around 68% of all patients with BTC, corresponding to various targeted therapies including immune-checkpoint inhibitors and molecular targeted agents. In one recent study, the overall response rate (ORR) and DCR for patients with BTC receiving a variety of targeted therapies were reported as 33% and 88%, respectively [17].

The mechanistic basis of immunotherapy is that cancers utilize several mechanisms of immune escape to restrict or evade antitumor immune responses. MMR deficiency has been shown to accelerate the accumulation of genetic errors at microsatellites, leading to high levels of microsatellite instability (MSI-H). Several studies have demonstrated dMMR/MSI-H as an important predictive biomarker for treatment with immune-checkpoint inhibitors (ICIs) in all types of cancer patients, regardless of primary site [18, 19]. The TMB is another biomarker that is associated with a better response to immunotherapy [20]. Recently, the results of several clinical trials evaluating the efficacy and safety of immune-checkpoint inhibitors in biliary tract cancers were reported. In phase Ib (Keynote-028) and phase II (Keynote-158) studies, pembrolizumab provided durable antitumor activity in 6–13% of patients with advanced BTC and the response lasted at least 6 months, regardless of PD-L1 expression, with manageable toxicity [21]. Therefore, the current NCCN guidelines recommend pembrolizumab as a useful treatment option in certain circumstances (NCCN Guidelines Version 5.2020 Hepatobiliary Cancers). A recent phase 2 multi-institutional study investigated nivolumab in patients with advanced refractory BTC, of whom 59% (32/54) were diagnosed with iCCA [22]. The results showed that nivolumab was well tolerated and had modest efficacy with a durable response; the mPFS and median OS were 3.7 months and 14.2 months, respectively [22]. The efficacy of combined ICIs and chemotherapy regimens has also been reported. A single-arm, phase II trial reported that nivolumab plus gemcitabine/cisplatin offered promising efficacy (mPFS of 6.1 months and mOS of 8.5 months) for patients with advanced BTC [23]. A further phase II trial investigated the efficacy and safety of combined camrelizumab and gemcitabine/oxaliplatin regimens in patients with BTC (NCT03486678). The preliminary results showed that among the 37 assessable patients with BTC, the objective response rate was 54% (95% CI, 38 to 69), and disease control was reported in 33/37 (89%; 95% CI, 75 to 96) of the treated participants [24]. Several more clinical trials of ICIs alone or in combination with other therapies are planned or ongoing in BTC and CCA (Table 1).

**Table 1:** On-going clinical trials of immune checkpoint inhibitors, alone or in combination with other therapies, in hepatobiliary cancer

Phase	NCT number	Conditions	Interventions
II	NCT04238637	Intrahepatic cholangiocarcinoma	Durvalumab/tremelimumab + Y90
II	NCT03486678	Biliary tract cancer Cholangiocarcinoma	SHR-1210 + GEMOX
II	NCT03111732	Biliary tract neoplasms Cholangiocarcinoma Bile duct cancer	Pembrolizumab + CAPOX
III	NCT04003636	Biliary tract carcinoma	Pembrolizumab + GP/Placebo
II	NCT04057365	Biliary tract cancer	Nivolumab + DKN-01
II	NCT03092895	Advanced primary liver cancer Advanced biliary tract carcinoma	SHR-1210 + Apatinib/GEMOX/FOLFOX
II	NCT03704480	Advanced biliary tract carcinoma	Durvalumab + Tremelimumab with or without Paclitaxel
III	NCT03478488	Biliary tract neoplasms	KN035 + GEMOX
I/II	NCT03311789	Biliary tract cancer	PD-1 inhibitor + Gemcitabine + Cisplatin
III	NCT03875235	Biliary tract neoplasms	Durvalumab + GP/Placebo
II	NCT04704154	Solid tumor	Regorafenib + Nivolumab

Integrative molecular signature and gene profiling techniques conducted in biological samples from CCA patients have revealed oncogenic pathways which may be candidate targets for therapy. The Ras-MAPK pathway is one of the main signaling networks in CCA biology and has been correlated with cell survival. Several studies have shown that activation of the Ras-MAPK pathway and EGFR/HER2 signaling network in CCA is associated with poor prognosis [25, 26]. Therefore, tyrosine kinase inhibitors targeting these pathways may be attractive strategies to investigate for the treatment of CCA. However, results from early-phase clinical trials of MET or EGFR inhibitors including erlotinib, cabozantinib, and tivantinib in BTC have been disappointing, with limited activity and substantial toxicity [27, 28].

Fibroblast growth factor receptor 2 (FGFR2) fusion and rearrangements were recently identified almost exclusively in iCCA, occurring in about 10–16% of patients, and found to be targetable in a certain subset of patients [29, 30]. FGFR2 aberrations were usually associated with improved outcome. Pemigatinib, an oral inhibitor of FGFR1, 2, and 3, was recently approved by the FDA for treatment of patients with previously treated, unresectable, locally advanced, or metastatic CCA with FGFR2 fusion or rearrangement, based on the results of the FIGHT-202 trial [31, 32]. Infigratinib is an oral FGFR 1–3 kinase inhibitor that has shown favorable results in a phase II trial in patients with advanced/metastatic FGFR-altered cholangiocarcinoma [33]. A phase III, multicenter, open-label, randomized trial (PROOF 301; NCT03773302) of infigratinib in comparison to gemcitabine/cisplatin in patients with advanced or metastatic CCA with FGFR2 translocations is ongoing. Futibatinib (TAS-120), a highly selective pan-FGFR inhibitor, has shown activity against FGFR2 resistance mutations. Results from an early-phase study of futibatinib revealed clinical activity in patients with progressed FGFR-aberrant iCCA [34]. Finally, the pan-FGFR inhibitors NVP-BGJ398 and erdafitinib have also demonstrated impressive antitumor activity in patients with advanced-stage CCA harboring FGFR alterations, with a DCR of 82% and manageable toxicity (phase II: NCT02150967, phase I: NCT01703481) [33, 35].

Aberrations in IDH have also been detected in iCCA. *IDH1* and *IDH2* gene mutations were recently reported to be fairly specific to iCCA (10–23%) [36, 37]. Furthermore, the product of enzymatic activity of IDH1/2 can be detected in serum, which may be a promising potential biomarker [38]. Ivosidenib (AG-120), an oral IDH1 inhibitor, has shown encouraging efficacy in patients with advanced *IDH1*-mutant, chemotherapy-refractory CCA [39, 40]. Remarkably, although the ORR of ivosidenib was only 5%, patients receiving this drug achieved a relatively long progression-free survival (21.8% at 12 months) and good tolerability (5% grade ≥ 3 toxicities). Based on these positive findings, other novel IDH inhibitors such as dasatinib and olaparib are being investigated in clinical trials. Furthermore, a phase I study investigating the combination of ivosidenib and cisplatin/gemcitabine in patients with advanced CCA is planned (NCT04088188).

Recently, CCA has been proposed to represent an epigenetically inclined mutational spectrum. For example, deficiencies in *ARID1A* and *PBRM1* expression have been associated with advanced-stage CCA [41]. Several preclinical and clinical studies of small-molecule inhibitors targeting chromatin-remodeling proteins are being investigated in CCA, including histone deacetylase inhibitors, such as vorinostat, romidepsin, and valproic acid, and DNA methyltransferase inhibitors, including azacytidine and decitabine. Valproic acid in particular has shown promising anti-tumor activity [42, 43]. Furthermore, mesothelin, a cell-surface protein, is often aberrantly expressed in CCA and associated with metastasis [44]. A phase I trial (NCT03102320) of anetumab ravtansine is currently enrolling patients with advanced-stage CCA with aberrant mesothelin expression. Somatic mutations of the tumor-suppressor genes *BRCA1* and *BRCA2* have also been reported in CCA. *BRCA*-mutant tumors are demonstrated to be sensitive to PARP inhibition. According to a retrospective clinical analysis, one of four CCA patients who received PARP inhibitor treatment achieved a PFS of 42.6 months.

Vascular endothelial growth factor (VEGF) overexpression is a poor prognostic factor in iCCA [45]. However, targeting VEGF has not produced satisfactory outcomes so far. Multikinase VEGF receptor inhibitors including sorafenib, lenvatinib, and regorafenib, which has proved positive efficacy in HCC, have reported disappointing results in iCCA [46–48]. Despite the unsatisfactory efficacy of VEGF-inhibitor monotherapy, alternative strategies such as lenvatinib combined with pembrolizumab have shown encouraging preliminary results. A single-arm study evaluated the efficacy and safety of second-line and beyond lenvatinib plus pembrolizumab in patients with refractory BTC; the ORR was 25%, the DCR was 78.1%, mPFS was 4.9 months, and mOS was 11.0 months [49]. The latest reports from ESMO 2020 have also shown encouraging survival benefits for the anti-PD1 agent toripalimab and lenvatinib in combination with oxaliplatin and gemcitabine (GEMOX) chemotherapy [50]. The ORR was 80%, and the DCR was 93.3%; furthermore, ORR was significantly associated with PD-L1 expression and DNA damage repair–related mutations in tumor samples [50]. These results suggest that immunotherapy/targeted therapy combinations are a promising strategy for the treatment of CCA.

NTRK fusions, which have been identified in 3.5% of patients with iCCA, are a further potential therapeutic target [32]. Larotrectinib and entrectinib are currently approved first-generation tropomyosin receptor kinase inhibitors and have reported an impressive ORR of 57% to 75% in advanced solid tumors harboring NTRK fusions [51, 52]. Although gene-targeted drugs have brought promising therapeutic benefit, unfortunately, curative therapies still require a lot of time for full development. The genetic heterogeneity of iCCA and associated rapid development of drug resistance are the possible causes for this [53].

## Summary

In this case, a middle-age male patient was diagnosed with iCCA by post-operative pathology. The patient received repeated radical resection and standard adjuvant or systemic chemotherapy. After experiencing failed locoregional therapy (two sessions of TACE and one course of microwave ablation), he was given combination treatment with camrelizumab plus apatinib at the third recurrence. The patient achieved a rapid CR that lasted for 8 months. However, increasing the dose interval of camrelizumab from twice a week to three times a week resulted in tumor progression. After returning to twice-weekly camrelizumab, the patient regained disease control for another 5 months. Finally, tumor progression was detected again accompanied by elevated CA 19.9 levels. A tumor biopsy was performed to evaluate NGS. Although PD-L1, TMB, and dMMR/MSI-H were detected at low levels in tumor tissue, this patient still benefited from combined treatment with immune checkpoint inhibitors and lenvatinib and regained disease control. It should be noted that the *IDH1* inhibitor ivosidenib has not been approved in China and was not available for this patient. Apatinib and lenvatinib both worked well in the treatment procedure.

The advancement of genomic profiling techniques has helped unravel the heterogeneity of iCCA and identify targetable molecular alterations. Several clinical trials of targeted therapies and immune-therapies in iCCA have already produced promising early results in the refractory setting. However, almost all patients will eventually develop treatment resistance. Therefore, repeated profiling may be valuable and help reveal new targets. Unfortunately, a large group of patients with iCCA do not harbor any known targetable genomic alterations, as reflected in our case. Assessing if an individual patient will benefit from targeted or immunotherapy should be considered carefully by a multidisciplinary team of scientists and clinicians. Meanwhile, it is imperative to further explore and understand the complex crosstalk among the oncogenic pathways in iCCA. Further studies should be launched to explore more possible therapeutic approaches for iCCA.
